# Reprogramming and transdifferentiation for cardiovascular development and regenerative medicine: where do we stand?

**DOI:** 10.15252/emmm.201504395

**Published:** 2015-07-16

**Authors:** Antje D Ebert, Sebastian Diecke, Ian Y Chen, Joseph C Wu

**Affiliations:** 1 Stanford Cardiovascular Institute, Stanford University School of MedicineStanford, CA, USA; 2 Department of Medicine, Division of Cardiology, Stanford University School of MedicineStanford, CA, USA; 3 Institute of Stem Cell Biology and Regenerative Medicine, Stanford University School of MedicineStanford, CA, USA; 4 Max Delbrück CenterBerlin, Germany; 5 Berlin Institute of HealthBerlin, Germany

**Keywords:** cardiomyocytes, disease modeling, genome editing, human induced pluripotent stem cells, tissue engineering

## Abstract

Heart disease remains a leading cause of mortality and a major worldwide healthcare burden. Recent advances in stem cell biology have made it feasible to derive large quantities of cardiomyocytes for disease modeling, drug development, and regenerative medicine. The discoveries of reprogramming and transdifferentiation as novel biological processes have significantly contributed to this paradigm. This review surveys the means by which reprogramming and transdifferentiation can be employed to generate induced pluripotent stem cell-derived cardiomyocytes (iPSC-CMs) and induced cardiomyocytes (iCMs). The application of these patient-specific cardiomyocytes for both *in vitro* disease modeling and *in vivo* therapies for various cardiovascular diseases will also be discussed. We propose that, with additional refinement, human disease-specific cardiomyocytes will allow us to significantly advance the understanding of cardiovascular disease mechanisms and accelerate the development of novel therapeutic options.

## Introduction

Despite advances in medical therapy, cardiovascular disease (CVD) remains a leading cause of morbidity and mortality worldwide. Concerted efforts in fundamental and translational research are required to provide novel diagnostic tools and effective therapeutic approaches for CVD. Mechanistic modeling of CVD as well as preclinical validation of therapeutic strategies will assist in the development of next-generation medical therapies that incorporate recent discoveries in stem cell biology.

## Human induced pluripotent stem cell-derived cardiomyocytes as a novel platform

**Figure 1:**
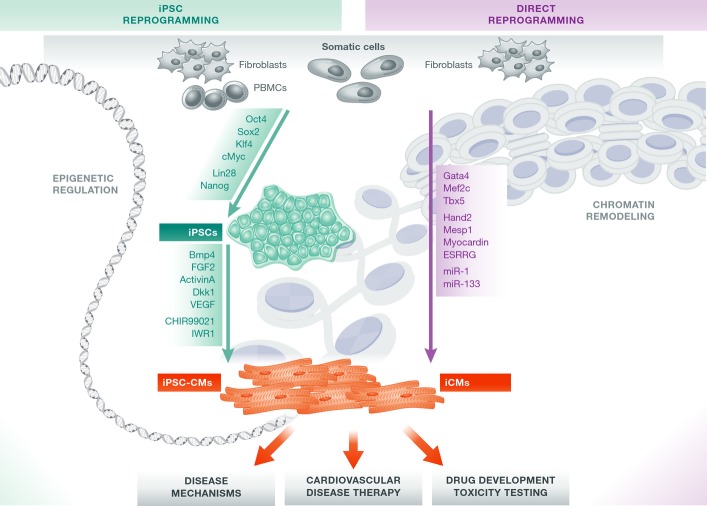
Generation and applications of patient-specific cardiomyocytes From isolated patient-specific source cells such as dermal fibroblasts or peripheral blood mononuclear cells (PBMCs), cardiomyocytes can be generated via iPSC reprogramming and subsequent differentiation to iPSC-CMs, or by transdifferentiation into iCMs. Both strategies employ a set of defined factors that cause drastic modulatory changes in the cellular epigenome. Disease-specific mutations within iPSCs can be corrected via genome editing approaches and can be employed for studying disease mechanisms, drug discovery, and regenerative medicine. While *in vivo* applications of iCMs are already being evaluated, the suitability of iCMs for other purposes such as disease mechanism and drug development studies remains to be ascertained.

Although animal models have provided indispensable insights into systemic whole-organ function *in vivo* as well as *in vitro* disease mechanisms (Fiedler *et al*, 2014; Houser *et al*, 2012; Duncker *et al*, 2015), not all findings from research on rodent cardiomyocytes can be translated to human cardiomyocytes at the cellular and molecular levels. Human cardiomyocytes, on the other hand, are a limited resource and cannot be indefinitely maintained in culture. These facts emphasize the need for novel human cellular and physiological models of CVD. Over the past decade, rapid technological advances have combined medical and basic sciences in the development and evaluation of novel therapeutics. One exciting advance has been the ability to generate patient-specific induced pluripotent stem cells (iPSCs; Takahashi & Yamanaka, 2006). Human iPSCs resemble human embryonic stem cells (ESCs), the “gold standard” for pluripotency, in their biological properties but without the ethical and political concerns associated with the use of human embryos. Therefore, iPSCs and their differentiated cardiomyocytes (iPSC-CMs) are considered a viable new and ethically less problematic, alternative platform for studying mechanisms of CVD and evaluating novel therapeutic avenues (Fig1). In addition, human iPSCs present the unprecedented opportunity to study disease-specific differences in a patient-specific manner, taking into account indivi-dual drug responses within a patient population. The validity of this approach is exemplified by the successful application of human iPSCs to model LEOPARD syndrome (Carvajal-Vergara *et al*, 2010), Timothy syndrome (Yazawa *et al*, 2011), long QT syndrome (Moretti *et al*, 2010; Itzhaki *et al*, 2011; Wang *et al*, 2014), arrhythmogenic right ventricular dysplasia (ARVD) (Kim *et al*, 2013; Asimaki *et al*, 2014), familial dilated cardiomyopathy (DCM; Sun *et al*, 2012), familial hypertrophic cardiomyopathy (HCM; Lan *et al*, 2013), viral cardiomyopathy (Sharma *et al*, 2014) and aldehyde dehydrogenase 2 genetic polymorphism (Ebert *et al*, 2014). These accomplishments demonstrate the tremendous power and versatility of iPSC-CMs in helping to develop novel therapeutic approaches for CVD and paving innovative avenues for precision medicine in the future.

## Reprogramming of somatic cells to iPSCs

The initial proof-of-concept studies on generation of ESC-like cells were performed using retroviral transduction of mouse fibroblasts with the transcription factors Oct4, Sox2, Klf4, and c-Myc (Takahashi & Yamanaka, 2006). These first-generation iPSCs featured unlimited self-renewal, differentiation into tissues of all germ layers, and the ability to generate an entire organism. However, these earlier approaches involved random insertion of reprogramming factors into the cellular genome, with consequent risk of oncogenic transformation. Subsequently, newer and safer non-integrating approaches employing Sendai virus (Ban *et al*, 2011), adenovirus (Fusaki *et al*, 2009; Zhou & Freed, 2009), episomal plasmids (Okita *et al*, 2008; Yu *et al*, 2009), minicircle (Jia *et al*, 2010) or co-MIP (Diecke *et al*, 2015), mRNA (Warren *et al*, 2010) or microRNAs (Lin *et al*, 2011), and direct protein delivery (Kim *et al*, 2009) have been developed (Fig1). Based on initial observations in mouse ESCs, two states of pluripotency were defined, an earlier one occurring in normal embryonal development termed “naïve” versus a “primed” state (Nichols & Smith, 2009). Naïvety is the ground state of pluripotency. Naïve pluripotent stem cells (PSCs) can be maintained *in vitro* by supplying leukocyte inhibitory factor (LIF) combined with inhibition of MAPK/ERK kinase (MEK) and glycogen synthase kinase 3 (GSK3) signaling and are characterized by two active X chromosomes in female lines. Primed PSCs are dependent on fibroblast growth factor 2 (FGF2) signaling and transforming growth factor-β (TGFβ) signaling and display inactivation of one X chromosome (Nichols & Smith, 2009; Hirai *et al*, 2012). Human ESCs and iPSCs are consi-dered to share some properties of naïve mouse ESCs, but mainly resemble primed murine epiblast stem cells (Nichols & Smith, 2009; De Los Angeles *et al*, 2012). Naïve human iPSCs can be derived by reversion of primed iPSCs into a state that resembles naïve mouse ESCs (Gafni *et al*, 2013; Theunissen *et al*, 2014). Currently, it is unknown whether these naïve human ESCs and iPSCs represent distinct intermediates in embryonic development. Further research is required to elucidate whether human naïve iPSCs may be more amenable to introduction of genomic modifications (Gafni *et al*, 2013) or may differentiate more efficiently into somatic tissues (Honda *et al*, 2013; Rais *et al*, 2013; Theunissen *et al*, 2014).

It has been acknowledged that reprogrammed iPSCs can retain specific DNA methylation profiles associated with their parental source cell type (Bar-Nur *et al*, 2011; Kim *et al*, 2011; Lister *et al*, 2011). Variations in these signatures also appear to account for intra-line variability among different clones originating from the same iPSC line (Kim *et al*, 2011; Lister *et al*, 2011). The long-term effect of epigenetic pattern retention, such as methylation profiles from the originating somatic cell type, is not yet fully understood. However, the somatic source cell type is known to affect differentiation efficiency into iPSC-CMs. For example, cardiac progenitor cell-derived iPSC lines have shown an enhanced ability to differentiate into cardiomyocytes compared to fibroblast-derived iPSC lines (Sanchez-Freire *et al*, 2014). Prolonged propagation of iPSCs through many passages reduces these effects, suggesting that residual epigenetic memory is attenuated in the course of long-term culture (Ohi *et al*, 2011; Sanchez-Freire *et al*, 2014). These studies demonstrate that epigenetic memory is a key determinant of iPSC differentiation into lineages that are distinct from the parental cell type.

Pluripotent reprogramming and transdifferentiation of cells from one germ layer to another (Ladewig *et al*, 2013) have altered the concept of cell fate as determined by unidirectional progression (Takahashi & Yamanaka, 2006; Ieda *et al*, 2010; Vierbuchen *et al*, 2010; Huang *et al*, 2011) and illustrate the plasticity of differentiation and lineage specification. Epigenetic roadblocks repressing chromatin in its inactive state occur during iPSC reprogramming (Kim *et al*, 2010; Carey *et al*, 2011; Theunissen & Jaenisch, 2014), and a similar role for chromatin remodeling complexes also exists during cardiovascular development (Chang & Bruneau, 2012; Bevilacqua *et al*, 2014). Epigenetic modulators can alter DNA methylation and histone acetylation profiles, thereby opening or repressing chromatin in target gene loci that direct lineage fate. Clearly, epigenetic checkpoint regulators of development and lineage differentiation (Takeuchi & Bruneau, 2009; Wang, 2012) are candidates for targeted modulation during iPSC reprogramming and cardiac differentiation. In this context, small molecule compounds are a highly promising resource for further improving the efficiency of cardiovascular lineage differentiation (Efe *et al*, 2011).

## Cardiac differentiation of iPSCs

Today, efficient differentiation protocols allow the generation of large quantities of highly enriched cardiomyocyte populations. These achievements have been made possible by pivotal work utilizing spontaneous aggregation of iPSCs in suspension as embryoid bodies (EBs) in combination with stage-defined growth factors (Kehat *et al*, 2001; Mummery *et al*, 2007; Burrdige *et al*, 2011; Kattman *et al*, 2011). Subsequently, these protocols led to the establishment of monolayer systems that stimulate the superfamily of TGFβ receptors via growth factors such as activin A and bone morphogenetic protein 4 (BMP4; Paige *et al*, 2010; Zhang *et al*, 2012), resulting in activation and repression of the canonical Wnt signaling pathway, respectively. Most recently, specific small molecules have been employed to replace growth factors as modulators of these signaling pathways (Fig1; Lian *et al*, 2012).

The relative immaturity of iPSC-CMs remains a challenge because it limits their use for disease modeling, drug discovery, and regenerative medicine purposes (Karakikes *et al*, 2015). Attempts to bypass this limitation have demonstrated that long-term culture enhances the appearance of more mature sarcomeric structural organization in iPSC-CMs (Kamakura *et al*, 2013). In addition, external cues such as electrical stimulation and mechanical cyclic stretching have been reported to aid in obtaining functionally mature iPSC-CMs (Lieu *et al*, 2013; Hirt *et al*, 2014a). Improvements in iPSC-CM maturation were also achieved via novel 3D culture methods (Nunes *et al*, 2013; Rao *et al*, 2013) and genetic overexpression of distinct factors (Fu *et al*, 2011; Bett *et al*, 2013; Lieu *et al*, 2013), and these approaches are currently subjects of intense research.

Maturation affects specification of cardiomyocyte subtypes and is vital for accurate recapitulation of disease phenotypes (Lan *et al*, 2013), including fundamental functional features such as more mature ion channel currents, densities, and kinetics (Sartiani *et al*, 2007; Yang *et al*, 2014). The early developmental stage produced by state-of-the-art iPSC-CM differentiation protocols is sufficient for analysis of certain hereditary channelopathies that cause ventricular tachyarrhythmias (Sallam *et al*, 2015). However, depending on the exact stage of development, there are numerous differences in electrical conduction and coupling as well as the contractile rate and force, compared to human adult cardiomyocytes (Karakikes *et al*, 2015). Furthermore, insufficient maturation of iPSC-CMs compared to adult human cardiomyocytes has also hindered a broader application of these cells for primary drug discovery and validation. Nevertheless, their use in exploratory studies and for examination of drug toxicity is clearly justified.

A related concern regarding iPSC-CM differentiation is the varying degree of heterogeneity achieved in the generated cardiomyocyte population. Current iPSC or ESC differentiation strategies yield a heterogeneous mixture of atrial-like and ventricular-like lineages, as well as pacemaker-like lineages such as atrioventricular node-like, sinoatrial node-like, and Purkinje fiber-like cells (Burridge *et al*, 2012). A deeper understanding of directed lineage differentiation, followed by its modulation, would facilitate subtype-specific cardiac differentiation. This can include direct manipulations at the epigenetic level or by achieving mRNA-based delivery of lineage-specific factors (Ong *et al*, 2015).

The most immediate need, however, is to achieve defined culture conditions and standardized protocols that address the issue of iPSC-CM maturation. In a broader sense, reproducibility and standardization throughout the scientific community will be a key to ensuring comparable datasets, as well as strides toward a broader applicability of iPSC-CMs for disease modeling and drug development. Although still at its infancy, the field has already made significant progress toward the defined derivation and propagation of human iPSCs and iPSC-CMs (Chen *et al*, 2011; Burridge *et al*, 2014; Ribeiro *et al*, 2015).

## Genetic engineering and personalized medicine

To understand the molecular and genetic determinants of CVD, advanced genome editing techniques are required to study genotype/phenotype relationships and to allow for the correction of patient-specific mutations in human iPSCs (Wang *et al*, 1995; Chen *et al*, 1998; Schwartz *et al*, 2000; Benson *et al*, 2003; Fig1). Initial pioneering work was performed using zinc-finger nucleases (ZFNs), a widely used technology for genomic correction that relies on the fusion of the FokI restriction endonuclease with zinc-finger proteins. These nucleases induce target site-specific double-stranded breaks, which stimulate endogenous DNA repair pathways. Due to the complexity of the required engineering steps, ZFNs have been largely supplanted by transcription activator-like effector nucleases (TALENs), and more recently by the clustered regulatory interspaced short palindromic repeats (CRISPRs)/Cas9 nuclease system. TALENs display enhanced specificity as well as reduced off-target action compared to ZFNs. Importantly, single-base pair recognition by TALENs or CRISPRs can correct single nucleotide exchange mutations (Hockemeyer *et al*, 2011; Ding *et al*, 2013; Lin *et al*, 2014). CRISPRs are the most accessible means to facilitate and optimize genetic engineering. Their specificity and off-target effects are currently being evaluated, as these nucleases have the potential to bind and cut sites other than the primary target site (Hendel *et al*, 2015). Nevertheless, in a relatively short time, CRISPRs have been demonstrated to be a cost-effective and time-efficient approach for genomic correction or introduction of site-specific mutations (Sander & Joung, 2014). Genome-corrected and disease-introduced isogenic cell lines are particularly valuable, as they share a common genotype with the exception of the disease-causing mutation, thereby eliminating confounding effects from genetic heterogeneity. Genomic modification to directly correct disease-specific point mutations *in vitro* is also valuable for exploring drug development in patient-specific cardiomyocytes. Human iPSC-CMs are currently being utilized as a system to evaluate novel and existing medications and to test patient-specific drug responses (Liang *et al*, 2013; Navarrete *et al*, 2013; Wang *et al*, 2014). For instance, iPSC-CMs from patients carrying long QT syndrome mutations (e.g., KCNQ1 G269S) and genome-edited iPSC-CMs with these disease-causing mutations were both shown to display long QT phenotypes (Liang *et al*, 2013; Wang *et al*, 2014). Furthermore, both cell types revealed comparable disease-specific responses following drug treatment (e.g., nifedipine) to rescue prolongation of action potential duration (APD) (Liang *et al*, 2013; Matsa *et al*, 2014; Wang *et al*, 2014). Overall, these examples illustrate the potential for using genome editing to generate accurate, reliable, and less expensive *in vitro* human models for understanding CVD and for accelerating drug discovery (Fig1; Ebert *et al*, 2012). Moreover, genome editing may accelerate the future clinical application of integration-free cell-based gene therapy, including the autologous transplantation of patient-specific, genome-corrected iPSC-CMs.

The complexity of genotype/phenotype relationships is further magnified by genetic background variation and variability among iPSC lines (Table1). Genome-wide association studies (GWAS) and subsequent data mining identify signaling pathways governing the control of disease-relevant targets. Large numbers of critical gene loci and related mutations have been described by GWAS and linked to pathogenic phenotypes. Variants occurring in these regions can influence the regulation of disease-relevant gene expression (Fig1). Moreover, late-onset or incomplete penetration of the disease phenotype can complicate further readout and genotypic correlation. In those cases, response profiling of well-established compounds and drugs in CVD might provide further insight. However, the presence of line-to-line and genetic background variation implies that additional layers of control are required to confirm genotype/phenotype relationships. Rescue of pathogenic functional features following genomic correction of the disease-related locus via TALENs or CRISPRs, the use of isogenic controls, and sufficiently powered studies are means to address these limitations. Given our ability to introduce specific disease-causing mutations into both iPSCs and ESCs, it is likely that instead of isolating primary cells from affected patients and generating disease-specific iPSCs and iPSC-CMs, the field will evolve toward standardized procedures based on introducing mutations of interest into fully sequenced and characterized reference stem cell lines (iPSCs and/or ESCs) to assess disease-specific genotypic and phenotypic relationships (Sallam *et al*, 2015).

**Table 1:** Challenges and opportunities of *de novo* generated cardiomyocytes for disease modeling, drug discovery, and regenerative therapies

Parameters	CM generation strategy		
	iPSC reprogramming and differentiation	Direct reprogramming	Human ESC differentiation
Mechanism	De-differentiation to iPSCs followed by specific differentiation to CMs	Transdifferentiation	Specific differentiation to CMs
Timeline	2–3 months	2–3 weeks	2–3 weeks
Efficiencies (% cTnT)	90–95%	9–13%	90–95%
Genome editing, isogenic controls	Yes	No	Yes
Genetic variation	Yes	Not yet determined	No
Disease modeling, drug development	Yes	Current efficiencies too low	Yes
Patient-specific assessment of phenotypes and drug function	Yes	Currently undergoing investigation	No
*In vivo* preclinical evaluation of regenerative therapies	Yes	Yes	Yes
Clinical safety and efficacy	Not yet determined	Not yet determined	Currently undergoing investigation
Ethical concerns	No	No	Yes

## Direct conversion to induced cardiomyocytes (iCMs)

There are both advantages and disadvantages in reprogramming of somatic cells to iPSCs. The intrinsic properties of iPSCs enable the use of tools such as genome editing to facilitate our understanding of basic disease mechanisms, as well as to evaluate precision medicine approaches (Wilson & Wu, 2015). Nevertheless, despite metho-dological advances, the entire process of generating patient-specific iPSC-CMs still requires several months and presents a potential risk of teratoma formation for regenerative medicine, given that the presence of residual pluripotent cells in the final product cannot be completely excluded (Lee *et al*, 2013). As a result, other approaches that eliminate the need for pluripotent stem cell generation are being explored.

In recent years, proof-of-concept studies have shown that somatic cells can be directly converted to cardiomyocytes (Fig1; Ieda *et al*, 2010; Efe *et al*, 2011; Qian *et al*, 2012). Transgenic expression of three cardiac-specific transcription factors (Gata4, Mef2c, and Tbx5) resulted in the transdifferentiation of murine fibroblasts into contracting cardiomyocytes referred to as induced cardiomyocytes (iCMs; Ieda *et al*, 2010). Intriguingly, the same outcome has been observed during an epigenetic activation phase in the early stage of reprogramming. This approach employed ectopic expression of transcription factors together with growth factors (Efe *et al*, 2011), demonstrating elegantly how cellular complexity can be harnessed for the understanding of specific molecular processes. Other reports have shown that direct reprogramming of somatic cells to iCMs is also feasible using various small molecules and miRNAs (Jayawardena *et al*, 2012, 2014; Protze *et al*, 2012).

Recently, direct reprogramming of human fibroblasts has also been achieved (Islas *et al*, 2012). Several studies showed that the murine direct reprogramming factors Gata4, Mef2c, and Tbx5 (GMT), or GMT plus Hand2 (GHMT), were insufficient to transform human fibroblasts into iCMs (Nam *et al*, 2013; Wada *et al*, 2013), indicating that the differences between mouse and human cardiovascular development need to be considered for optimal transdifferentiation to human iCMs. A combination of Gata4, Hand2, Tbx5, myocardin, miR-1, and miR-133 could convert human adult dermal fibroblasts into induced cardiomyocyte-like cells (iCMLs). The transduction of these factors promoted substantial cardiac troponin T expression in at least 9% of the source population (Nam *et al*, 2013). Shortly afterward, introduction of GMT plus Mesp1 and myocardin (GMTMM) was also shown to successfully convert human fibroblasts to iCMLs (Wada *et al*, 2013). Since then, alternative approaches have succeeded in generating human iCMs with gene expression profiles and functional characteristics similar to those detected in ESC-CMs (Fu *et al*, 2013).

## Current limitations and the routes toward therapeutic application

Direct reprogramming as an alternative to deriving human iPSCs offers the advantage of a 2-week timeline, compared to 2–3 months (Table1). However, current methods for producing iCMs suffer from low efficiencies compared to iPSC differentiation (Chen *et al*, 2012). Depending on the combination of transcription factors used, human fibroblasts convert into iCMs with different efficiencies, ranging from 5% (GMTMM; Ieda *et al*, 2010) to 13% (Gata4, Hand2, Tbx5, Myocd, miRNA 1, and miRNA 133; Fu *et al*, 2013) based on troponin T-positive cells. Recently, polycistronic vectors have been used to express the GMT factors in appropriate stoichiometry as a single mRNA, which has significantly increased the efficiency of mouse fibroblast conversion *in vitro* up to 25% (Inagawa *et al*, 2012; Wang *et al*, 2015). However, these results remain to be replicated in human fibroblasts. In addition, successful generation of pure iCM populations has not yet been reported. Indeed, direct transdifferentiation has so far generated heterogeneous populations of cardiomyocyte-like cells representing various early developmental stages, which rarely display spontaneous beating and produce only sporadic action potentials (Fu *et al*, 2013). In general, the overall low transdifferentiation efficiency of iCMs into *bona fide* cardiomyocytes is the main obstacle for the required scale-up of cell production.

Like iPSC-CMs, iCMs must undergo additional maturation before they can serve as true models of adult cardiomyocytes (Bedada *et al*, 2014; Yang *et al*, 2014; Jayawardena *et al*, 2015). The accurate determination of the differences between these cell types requires the direct comparison of iCMs with both iPSC-CMs and human adult cardiomyocytes (Protze *et al*, 2012; Wada *et al*, 2013). While similarity of iCMs to ESC-CMs (Fu *et al*, 2013) has been reported, including subtype specification as a feature of mature cardiomyocyte populations (Nam *et al*, 2014), other studies have indicated that human iCMs generated *in vitro* may be even more immature than human iPSC-CMs (Wada *et al*, 2013). These findings imply that iCMs may reflect the early fetal stage of embryonic cardiomyocytes, and hence, co-stimulation with appropriate factors may drive maturation of iCMs *in vitro*. Potential approaches include mechanical stimuli or secreted molecules related to the normal myocardial environment, such as cardiac matrix scaffolds and secreted paracrine factors. Exposure to stretching forces is also thought to accelerate iCM generation and maturation (Qian & Srivastava, 2013). Thus, current limitations of efficient generation and maturation of iCMs *in vitro* may be addressed by advancing *in vivo* reprogramming instead.

miRNAs have been demonstrated to be sufficient for direct reprogramming to iCMs without addition of any transcription factors (Jayawardena *et al*, 2012). Current research has thus far focused on their use as powerful drivers of lineage fates (Cordes & Srivastava, 2009; Boon & Dimmeler, 2015). It is likely that miRNAs promote cardiac induction by suppressing fibroblast signatures, as for example, miRNA133-mediated inhibition of Snai1-controlled expression pathways (Muraoka *et al*, 2014). Therefore, an optimized cocktail to promote the generation of more mature iCMs may include specific miRNAs, as well as specific chemical epigenetic modulators. Should such strategies result in higher efficiency of iCM reprogramming and improved maturation, safety and efficacy would need to be assessed in studies similar to those required for human iPSC-CMs. For now, a direct comparison of iCMs with iPSC-CMs regarding their functional properties remains to be performed. Initial comparative evaluations should focus on functional parameters such as electrophysiology and calcium handling, and also gene and protein expression patterns. Subsequently, the beneficial effects of either cell type on improving cardiac function in preclinical models of CVD will need to be demonstrated. Likewise, the long-term stability of iPSC-CM and iCM phenotypes must be assessed to address safety and efficacy issues.

## Heart disease and novel therapeutic approaches

**Figure 2:**
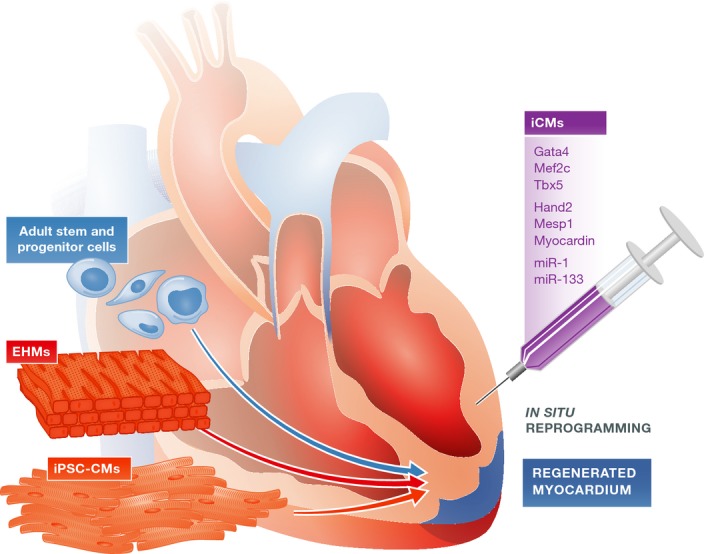
Cell therapy and tissue engineering approaches for cardiovascular disease therapy Heart failure due to ischemic heart disease or genetic disorders remains a major healthcare burden. Potential novel treatment options include transplantation of iPSC-CMs or ESC-CMs, as well as direct *in vivo* reprogramming of cardiac fibroblasts in the scar region to iCMs. The regenerative capacity of adult stem and progenitor cell populations is also being evaluated. Tissue engineering is a new method that aims to re-muscularize damaged myocardium via transplantation of *in vitro* engineered heart muscle made from iPSC-CMs or ESC-CMs.

Two of the main classes of genetically inherited heart diseases include channelopathies and cardiomyopathies. Channelopathies or arrhythmic cardiac disorders are caused by mutations in genes encoding ion channels, such as *SCN5A* that encodes the cardiac Na^+^ channel α-subunit. The functional characteristics include voltage gating and/or protein trafficking defects, which can result in gain or loss of function in the Na^+^ channel and subsequent ventri-cular arrhythmias, leading to diseases such as long QT syndromes (Lehnart *et al*, 2007). By contrast, cardiomyopathies, or defects in heart muscle contraction, are most frequently caused by mutations in cytoskeletal or contractile proteins (McNally *et al*, 2013). The relevant pathogenic features are transversely isotropic, consisting of irreversible ventricular dilatation and systolic dysfunction that cause severely impaired ventricular contraction. Both channelopathies and cardiomyopathies can also be caused by non-hereditary, acquired determinants such as chronic or acute ischemia (Fig2), and by drugs or autoimmune events (Kass, 2005).

Human models of iPSC-CMs have successfully recapitulated numerous genetically determined CVD, including long QT, DCM, HCM, and ARVD (Moretti *et al*, 2010; Yazawa *et al*, 2011; Davis *et al*, 2012; Sun *et al*, 2012; Caspi *et al*, 2013; Lan *et al*, 2013). These studies have demonstrated that iPSC-CMs display phenotypic disease features such as abnormal sarcomere alignment and striation, as well as critical functional properties such as propagation of calcium transients in amplitude, time to peak, duration, and decay (Moretti *et al*, 2010; Yazawa *et al*, 2011; Davis *et al*, 2012; Sun *et al*, 2012; Caspi *et al*, 2013; Lan *et al*, 2013). Importantly, iPSC-CMs allow for quantitative analysis of electrical properties regarding the action potential’s upstroke velocity, time to peak, and duration. For example, abnormally prolonged APD and decreased repolarization velocity are detected in long QT patient-derived iPSC-CMs (Yazawa *et al*, 2011; Table1).

## Pathogenesis of myocardial infarction and regeneration of the heart

The early phase during and after an infarction is characterized by inflammatory, necrotic, and apoptotic cellular responses. The ensuing late or chronic phase includes an expansion of the infarcted region in the myocardial wall, recruitment of myeloid cells, tissue necrosis, and degradation of the extracellular matrix (ECM). Subsequent neoangiogenesis and remodeling of the left ventricle (LV) entails scar formation, hypertrophic expansion of cardiomyocytes, and fibrosis. The resulting dilation of the LV is accompanied by increased frequency of arrhythmias, myocardial dysfunction, and eventually heart failure. Conventional therapy of fibrosis and LV dilatation by angiotensin converting enzyme (ACE) inhibitors and angiotensin II receptor blockers (ARBs) partially counteracts these deleterious consequences and attenuates adverse remodeling (Dorn, 2009). Nevertheless, viable cardiomyocytes are lost to a large extent in the area of myocardial infarction (MI).

Recent studies suggest that the heart is capable of limited endogenous regeneration (Bergmann *et al*, 2009; Parmacek & Epstein, 2009; Porrello *et al*, 2011; van Berlo *et al*, 2014). While proliferation of the heart may occur to a minor extent throughout the lifetime of an organism, active cell division of cardiomyocytes is limited to the embryonic stage (Bergmann *et al*, 2009; Porrello *et al*, 2011). A different source of endogenous heart regeneration is the resident adult stem cell population, known as cardiac progenitor cells (CPCs), reported to be capable of differentiating and proliferating to replenish apoptotic cardiomyocytes (Fig2; Dimmeler *et al*, 2005; Leri *et al*, 2011). The full regenerative capacities of these cells remain controversial and are discussed in detail elsewhere (Laflamme & Murry, 2011; Anversa *et al*, 2013; Maillet *et al*, 2013, van Berlo *et al*, 2014). Overall, the endogenous proliferation and repair abilities of the heart are not sufficient to allow the repopulation of damaged myocardial areas with new cardiomyocytes following MI.

## Stem cell-derived cardiomyocytes for heart disease therapy

Previous clinical trials have employed various adult stem cell and progenitor cell populations to test their efficacy for therapeutic applications (Fig2; Assmus *et al*, 2002; Schachinger *et al*, 2004; Losordo *et al*, 2007; Chugh *et al*, 2012; Hare *et al*, 2012; Makkar *et al*, 2012; Traverse *et al*, 2012; Vrtovec *et al*, 2013; Karantalis *et al*, 2014). Safety and feasibility of these cells have been demonstrated in these scenarios, and extensive efforts have been spent on exploring the therapeutic potential of these cells. Overall, the results have shown varying degrees of clinical benefit in MI patients (Sanganalmath & Bolli, 2013). Currently, additional approaches are being explored, including transplantation of new cell types (e.g., human ESC-cardiac progenitor cells (Menasche *et al*, 2015) or iPSC-CMs), or application of alternative delivery approaches such as implantation of *in vitro* constructed cell sheets of engineered heart muscles (EHMs) (Fig2; Zimmermann, 2013; Emmert *et al*, 2014; Hirt *et al*, 2014b). One mechanism by which cell therapy (e.g., ESC-CMs or iPSC-CMs) may improve outcomes is via engraftment of transplanted cells within the host environment, which in theory would lead to the replacement of damaged cardiomyocytes and fibrotic tissue, and restore structural support of the ventricular walls. Extensive studies have focused on grafts within the non-infarcted versus infarcted myocardium in small and large animal models (Laflamme *et al*, 2007; van Laake *et al*, 2008). Human grafts express cardiac markers and displayed sarcomere alignment as well as integration with the host’s vasculature. In this context, coupling of transplanted cardiomyocytes with the host myocardium is considered a vital factor contributing to improved cardiac function. Stem cell-derived cardiomyocytes couple to a limited extent with the host myocardial cells in small animal models (Kehat *et al*, 2004). Large animal models such as pigs and non-human primates are more valuable due to the greater resemblance of their heart rates with the beating frequency of transplanted cardiomyocytes (Chong *et al*, 2014). However, to a large extent, functional improvement in left ventri-cular ejection fraction (LVEF) in some of these transplantation models has been suggested to result through the release of paracrine factors (Gnecchi *et al*, 2005; Gu *et al*, 2012; Huber *et al*, 2013). Transplanted cells may secrete signaling molecules that exert beneficial functions directly or by altering gene expression patterns in the surrounding myocardium. Such paracrine mechanisms have been frequently proposed to contribute to the recovery of cardiac function (Gnecchi *et al*, 2005). Well-studied factors such as vascular endothelial growth factor (VEFG; Zangi *et al*, 2013) and thymosin β-4 (TB4; Smart *et al*, 2010, 2011) have been selectively characterized in murine infarct models for their capacity to mediate cardiac repair. Recently, targeted approaches to identify specific paracrine factors revealed novel paracrine-acting proteins that could improve tissue and heart function following MI (Korf-Klingebiel *et al*, 2015). In the future, cell-based therapies may benefit from these findings by integrating delivery of specific factors into the transplanted therapeutic composite. Moreover, complementing the cell mixture with iPSC-derived endothelial cells may lead to beneficial effects from the developed vasculature (Ye *et al*, 2014). Together, these synergistic approaches may help promote engraftment, vascularization, and structural integrity of the ventricular walls.

To date, three fundamental issues have slowed the clinical translation of iPSC-CMs or ESC-CMs: the risk of tumor formation, poor survival of transplanted cells, and the need for immunosuppression for allogeneic ESC and iPSC derivatives. The first obstacle includes both tumors potentially arising from random insertion of integrating reprogramming vectors, and the risk of teratomas arising from residual undifferentiated stem cells (Lee *et al*, 2009, 2013). Novel non-integrating iPSC reprogramming strategies may decrease risk, while increasing the efficiency of differentiation or the purity of the final cell product could minimize teratoma risk (Tang *et al*, 2011). The second major obstacle is acute donor cell death due to hypoxia, anoikis, and inflammation, as well as lack of blood supply (Li *et al*, 2009a,b; Liu *et al*, 2012; Nguyen *et al*, 2014). Hence, the majority of current cardiac cell therapies (both basic and clinical) appear to achieve beneficial effects without long-term persistence of the cells, presumably through the release of paracrine factors to the host heart prior to transplanted cell loss as described earlier (Gnecchi *et al*, 2005). Relating practical issues include generation of sufficient infarct sizes in the chosen species to induce a measureable decline in heart function without killing the animal. In some cases (e.g., guinea pigs and dogs), the collateral circulation is so high that meaningful infarcts cannot be generated by coronary artery ligation (Verdouw *et al*, 1998). These concerns are critical for investigational new drug (IND)-enabling large animal studies in the evaluation of the safety and efficacy of stem cell-derived therapies.

The third obstacle toward clinical applications of iPSC-CMs or ESC-CMs is the need for effective immunosuppression to reduce rejection in allogeneic settings, which can be daunting (Pearl *et al*, 2011, 2012). The use of more sophisticated immunosuppressive or tolerance induction strategies (Huber *et al*, 2013) as well as combinations of iPSC-CMs with potentially immunotolerant iPSC-derived mesenchymal stem cells (iPSC-MSCs) is being investigated (Lian *et al*, 2010). An alternative approach is the creation of human leukocyte antigen (immunosuppression HLA)-matched cell banks from healthy donors that contain selected iPSC lines with maximized HLA genotype overlap, which may minimize the need for immunosuppression (Taylor *et al*, 2012; Neofytou *et al*, 2015). However, an important caveat to this approach was recently found in the heterogeneity of human mitochondria and, specifically, mismatched mitochondrial antigens, which by themselves can trigger rejection in transplant models (Deuse *et al*, 2015).

## *In vivo* applications of iCMs

Direct application of iCM reprogramming *in vivo* may promote patient-specific precision therapy by reducing the accompanying costs and efforts, which are considerable with *in vitro* generation of patient-specific iPSC-CMs. Induced cardiac regeneration *in vivo* via iCMs might circumvent current unresolved issues in iPSC-CM therapy, such as poor survival and engraftment of transplanted cells. However, the degree of functional cardiac improvement resulting from *in situ* transdifferentiated iCMs is unknown, as is the extent of their coupling and integration within the host myocardium (Table1). Safety and potential off-target effects of iCM reprogramming cocktails have yet to be studied in detail, and the consequences of *in vivo* transfection of “off-target” cells such as endothelial, smooth muscle, or cardiac cells in the heart are also unknown and can be problematic. Finally, another consideration is the reproducibility of iCM generation using viral delivery approaches, which can lead to host immune response, as compared to non-viral or small molecule approaches, which may have poor pharmacokinetics *in vivo* (Chen *et al*, 2012). In summary, many challenges remain to be resolved before therapeutic application of iCMs in the clinic can even begin.

## Tissue engineering

Currently, heart transplantation is the only viable therapy for end-stage heart failure but remains problematic due to a chronic shortage of organ supply, as well as the persistent risk of immune rejection. An alternative strategy for regeneration of damaged myocardium is to exploit therapeutic cells such as iPSC-CMs for the construction of 3D structures *in vitro*, and subsequent transplantation of these engineered cardiac patches (Caspi *et al*, 2007; Tulloch *et al*, 2011; Kawamura *et al*, 2012). This technology is known as “tissue engineering” or generation of engineered heart muscle (EHM). Transplantation of a tissue patch/EHM ensures increased precision of delivery onto damaged myocardial areas, as well as full retention of transplanted material. EHM transplants may also allow direct substitution of scar tissue in the infarcted area with new, healthy cardiac muscle, minimizing long-term damage resulting from scar growth and ultimately reducing adverse remodeling and improving cardiac function (Fig2). Moreover, it is expected that 3D cardiac tissues may mature into more adult-like structures compared to single cardiomyocytes, which is considered essential for optimal integration into the host environment. Several key features of maturation, such as alignment, orientation, and binucleation of cardiomyocytes (particularly their sarcomeric structural organization), were found to be improved in engineered tissues (Zimmermann *et al*, 2002; Tiburcy *et al*, 2011; Zhang *et al*, 2013). The beneficial outcomes of tissue engineering-based therapy have been extensively demonstrated in small animal models (Naito *et al*, 2006; Zimmermann *et al*, 2006; Sekine *et al*, 2008; Tulloch *et al*, 2011) and are currently being tested in large animal models. Eventually, EHMs may facilitate patient-specific organ transplantation via *in vitro* generation of partial or whole-organ structures.

Significant potential problems, such as poor survival of transplanted EHMs and their problematic integration into the host myocardium, need to be resolved before human trials can commence. A recent study using mouse cardiac sliced tissue as a benchmark to validate and model tissue-engineered patches showed poor survival with > 400 μ?m thickness (Riegler *et al*, 2014). Detailed understanding of the complex molecular mechanisms that determine the engraftment of transplanted EHMs within the host will lead to better strategies to address these issues. Possible solutions include co-delivery of supporting scaffold matrices, pro-survival cocktails, and stimulation of host myocardium via specific chemical molecules. Standardization of protocols for EHM generation, performance, and maturation will be a crucial step before moving forward to clinical trials. The substantial progress made during the past decade holds promise for a future clinical translation of EHM technology (Tee *et al*, 2012; Sekine *et al*, 2013; Ye *et al*, 2013; Hirt *et al*, 2014b).

## Conclusions

Pluripotent stem cell-derived cardiomyocytes, induced cardiomyocytes, and engineered heart muscle present exciting new opportunities for the development of novel CVD treatments. While iCM production is currently being optimized, iPSC-CMs provide a state-of-the-art patient-specific model system to study disease mechanisms and develop new drugs. Future studies will have to ascertain whether ESC-CM-, iPSC-CM-, iCM-, or EHM-based transplantation can achieve sustained improvement of cardiac function. These synergistic, multidisciplinary approaches should improve understanding of the mechanisms governing cardiovascular health and disease at the molecular, cellular, and organ levels. Transformation of this knowledge into therapeutic strategies is the key to achieve the full potential of regenerative medicine and open a new era of advances in cardiovascular therapy.

**Pending issues**

Maturation of pluripotent stem cell-derived cardiomyocytes (e.g., iPSC-CMs or ESC-CMs) and iCMs, as well as defined populations of cardiomyocyte subtypes (e.g., atrial-, nodal-, and ventricular-like cells).

Standardization of efficient, reproducible, and defined culture conditions to generate comparable data.

Improving the conclusiveness of patient-specific iPSC-CM models with approaches to address line-to-line variability and genetic background variations via CRISPR-generated isogenic controls, or fully sequenced and characterized reference stem cell lines (either iPSCs or ESCs).

Optimizing efficiencies in direct reprogramming to iCMs *in vitro* and *in vivo*.

Assessing efficacy of ESC-CM-, iPSC-CM-, iCM-, and EHM-based therapies.

## Abbreviations

Cell therapy: Transplantation of therapeutic cell populations (e.g., adult stem cells or PSC-CMs) to ameliorate damage in the infarcted heart and improve cardiac function
Disease modeling: Understanding the functional and molecular causes of diseases by recapitulating their respective phenotypes in organisms (e.g., mouse, zebrafish) or tissue-specific cell models (e.g., human iPSC-CMs)
Drug discovery: Identifying new therapeutics by employing a wide range of scientific methods and model systems
Genomewide association study (GWAS): A whole-genome association study that assesses common genetic variants (e.g., single nucleotide polymorphisms) in defined populations of individuals
Genome editing: Modifying the DNA of a given genome in specific sites with genetically engineered nucleases
Investigational new drug (IND) application: Filing admission of research on a new drug, treatment, or patient population in human subjects with a regulatory agency (e.g., the US Food and Drug Administration)
Non-integrating reprogramming: De-differentiation of somatic cells to induced pluripotent stem cells (iPSCs) using reprogramming factors that do not integrate into the host cell genome
Paracrine mechanisms: Chemical signals secreted from one cell (e.g., growth factors or chemokines) to induce functional changes in nearby cells
Tissue engineering: A strategy to replace damaged myocardium via generation of engineered heart muscle (EHM), which is a functional tissue-like structure constructed *in vitro* from adult stem cells or PSC derivatives
Transdifferentiation: Direct somatic cell reprogramming from one germ layer of origin to another
